# Quality Assessment of Systematic Review of the Bariatric Surgery for Diabetes Mellitus

**DOI:** 10.1155/2019/9541638

**Published:** 2019-11-21

**Authors:** Xinye Jin, Jinjing Wang, Xueqiong Li, Ping An, Haibin Wang, Wenfeng Mao, Qi Zhou, Yaolong Chen, Jie Wang, Kang Chen, Yiming Mu

**Affiliations:** ^1^Department of Endocrinology, Chinese PLA General Hospital, Beijing 100853, China; ^2^Department of Endocrinology, Hainan Hospital of Chinese PLA General Hospital, Sanya 572013, China; ^3^Department of Endocrinology, Fifth Medical Center of Chinese PLA General Hospital, East Avenue, Beijing 100071, China; ^4^Department of Gerontology, First Affiliated Hospital of Kunming Medical University, Kunming 650031, China; ^5^Medicine School of Nankai University, Tianjin, China; ^6^Evidence-Based Medicine Center, School of Basic Medical Sciences, Lanzhou University, Lanzhou, China; ^7^Chinese GRADE Center, Lanzhou, China; ^8^WHO Collaborating Centre for Guideline Implementation and Knowledge Translation, Lanzhou, China

## Abstract

**Objective:**

Using the AMSTAR tool, this study evaluated the quality of systematic reviews (SRs) that assessed the efficacy of bariatric surgery in diabetic patients. We aimed to identify studies that can be used as clinical references.

**Methods:**

Medline (via PubMed), EMBASE, Epistemonikos, Web of Science, Cochrane Library, CBM, CNKI, and Wanfang Data were systematically searched from inception to December 31, 2017. Two reviewers independently selected SRs and extracted data. Disagreements were solved by discussions or through consultation with a third reviewer. Reviewers extracted data (characteristics of included SRs, e.g., publication year, language, and number of authors) into the predefined tables in the Microsoft Excel 2013 sheet. Data were visualized using the forest plot in RevMan 5.3 software.

**Results:**

A total of 64 SRs were included. The average AMSTAR score was 7.4 ± 1.7. AMSTAR scores of 7 (*n* = 21, 32.8%) and 8 (*n* = 14, 28.1%) were most common. The AMSTAR scores of SRs published before 2016 (*n* = 46, 71.9%) were compared with SRs published after 2016 (*n* = 18, 28.1%), and no significant differences were observed (MD = −0.79, 95% confidence interval (CI) -1.65-0.07, *P* = 0.07). For SRs published in Chinese (*n* = 17, 26.6%) compared to those published in English (*n* = 47, 73.4%), the AMSTAR scores significantly differed (MD = 0.21, 95% CI (-0.55, 0.97), *P* = 0.59). For SRs published in China (*n* = 33, 51.6%) compared to those published outside of China (*n* = 31, 48.4%), significant differences in the AMSTAR scores were observed (MD = 1.10, 95% CI (0.29, 1.91), *P* = 0.008). For SRs with an author number ≤ 6 (*n* = 31, 48.4%) compared to SRs with authors ≥ 6 (*n* = 33, 51.6%), no significant differences were observed (MD = −0.36, 95% CI (-1.22, 0.50), *P* = 0.41). For high-quality SRs published after 2016 (*n* = 11, 17.2%) compared to other SRs (*n* = 53, 82.8%), statistically significant differences were noted (MD = 1.75, 95% CI (1.01, 2.49), *P* < 0.00001).

**Conclusions:**

The number of SRs assessing the efficacy of bariatric surgery in diabetic patients is increasing by year, but only a small number meet the criteria to support guideline recommendations. Study protocols not being registered, grey literature not retrieved, incorporation of grey literature as exclusion criteria, and failure to evaluate publication bias and report a conflict of interest were the main causes of low AMSTAR scores.

## 1. Background

Since 1980, the human body mass index (BMI) has increased at a rate of 4 kg/m^2^ per decade, and obesity rates continue to rise [1–3]. Obesity is an important risk factor for diabetes [4, 5]. The effective control and treatment of diabetes is important to prevent diabetic complications and improve the long-term outcome of diabetic patients [6]. At present, bariatric surgery is one of the fastest-growing operative procedures performed worldwide, with an estimated >340,000 operations performed in 2011 [7]. While the absolute growth rate of bariatric surgery in Asia was 449 percent between 2005 and 2009 [8], the number of procedures performed in the United States appears to have plateaued at approximately 200,000 operations per year [9–11]. In this regard, several guidelines recommend bariatric surgery as a treatment option for obese diabetic patients [12–15].

In 2011, the Institute of Medicine (IOM) defined the guidelines as follows: Clinical practice guidelines are statements that include recommendations intended to optimize patient care that are informed by a SR of evidence and an assessment of the benefits and harms of alternative care options [16]. In 2014, the World Health Organization (WHO) guideline and development manual requested that recommendations require evidence based on time-efficient, high-quality systematic reviews (SRs) [17]. At present, guidelines recommend that bariatric surgery should be used to treat obese patients with diabetes, and several SRs have been published to verify its efficacy [18–22]. However, it is unclear as to whether the quality of these SRs is sufficient to support reliable evidence of recommendations according to the WHO guidelines. The Assessing the Methodological Quality of Systematic Reviews (AMSTAR) [23] is an internationally acknowledged tool for evaluating the quality of SRs. The purpose of this study was to use AMSTAR to assess the quality of SRs that measure the efficacy of bariatric surgery during the treatment of diabetes and to provide references for relevant guidelines.

## 2. Methods

### 2.1. Study Selection

Studies were included if they met the following inclusion criteria: (1) patients meeting the criteria for diabetes diagnosis, (2) SRs in which information retrieval (database, search strategy, time, etc.) was reported, (3) SRs in which the type of intervention was weight loss surgery, and (4) availability of all included full texts.

Exclusion criteria included (1) old versions of SRs, (2) SRs containing meeting abstracts and incomplete manuscripts, and (3) SRs not in Chinese or English language.

### 2.2. Literature Search

Electronic searches were performed in the Medline (via PubMed), EMBASE, Epistemonikos, Web of Science, Cochrane Library, CBM, CNKI, and Wanfang databases for relevant articles published up to December 31, 2017. Search strategies were developed for each database using index terms: “Perioperative Period,” “Perioperative Care,” “Surgical Procedures, Operative,” “perioperative,” “peri-operative,” “preoperative,” “pre-operative,” “postoperative,” “post-operative,” “pre-surgery,” “peri-surgery,” “post-surgery,” “intraoperative,” “intra-operative,” “surgical,” “diabetes mellitus,” “diabet∗,” “IDDM,” “NIDDM,” “MODY,” “T2DM,” “T2D,” “T1DM,” “T1D,” “SR,” “meta-analysis,” “meta-analysis,” “meta analyses,” “meta-analyses” (see Additional [Supplementary-material supplementary-material-1]).

### 2.3. Screening and Data Extraction

Two rounds of preliminary tests were conducted by two independent reviewers (Xinye Jin, Qi Zhou) prior to screening, in order to reach consistent screening criteria. Two reviewers (Xinye Jin, Qi Zhou) then double screened the titles/abstracts and full texts. Researchers extracted the data according to the predetermined information extraction table. The following data were extracted: publication year, journal, language, number of authors, country of the first author, number of studies included, type of studies included, sample size, number of databases, and grading standard of evidence. Two reviewers (Xinye Jin, Qi Zhou) independently conducted literature screening and data extraction. Disagreements were solved by discussions or through consulting a third reviewer.

### 2.4. Quality Assessment

Two reviewers (Xueqiong Li, Ping An) independently applied the AMSTAR tool to evaluate the methodological quality of the SRs (see Additional [Supplementary-material supplementary-material-1]). Any disagreements were resolved by discussions or through consulting a third reviewer. The AMSTAR included 11 items. The evaluation results of each item were “Yes,” “No,” “Cannot answer,” and “Not applicable.” “Yes” denoted that the SR fully meets the requirements of the item; “No” denoted that the SR partially or fully dissatisfied the requirements of the item; “Cannot answer” denoted that the SR lacks relevant information to judge the item; “Not applicable” denoted that the item was unsuitable for appraising the SR (for example, are the methods used to combine the study findings appropriate? does not apply when the SR does not conduct data synthesis of any included studies). When the evaluation result of an item was “Yes,” it was scored 1 point. For “No,” “Cannot answer,” and “Not applicable,” 0 points were given. The AMSTAR ranged from 0 to 11 points.

The methodological quality as judged by AMSTAR was classified as high (8-11 points), moderate (4-7 points), or low (≤3 points) [24, 25]. The effective time of the SRs was 5.5 years (i.e., the time before new evidence that could alter the results had emerged), and 23% of SRs had an effective time of within 2 years [26, 27]. The Cochrane collaboration requires that any Cochrane SR should be updated within two years. If it was not updated, reasons for this were required. According to the retrieval time, SRs published in 2016 and beyond maintained appropriate timeliness. SRs published on or after 2016 in which the AMSTAR score ≥ 9 were regarded as high quality and could be used to support relevant recommendations in the guidelines.

### 2.5. Data Analysis

We used Excel software to perform descriptive statistical analysis of the following data: the difference in AMSTAR scores according to publication year, language, number of authors, and country of the first author. AMSTAR scores were calculated as the sum of items that evaluation results were “Yes.” We used a random effects model to estimate RR (Risk Ratio) and draw forest plots by RevMan 5.3 software. A two-sided *P* value of ≤0.05 was considered statistically significant.

## 3. Results

### 3.1. Literature Retrieval and Screening Results

**Figure 1:**
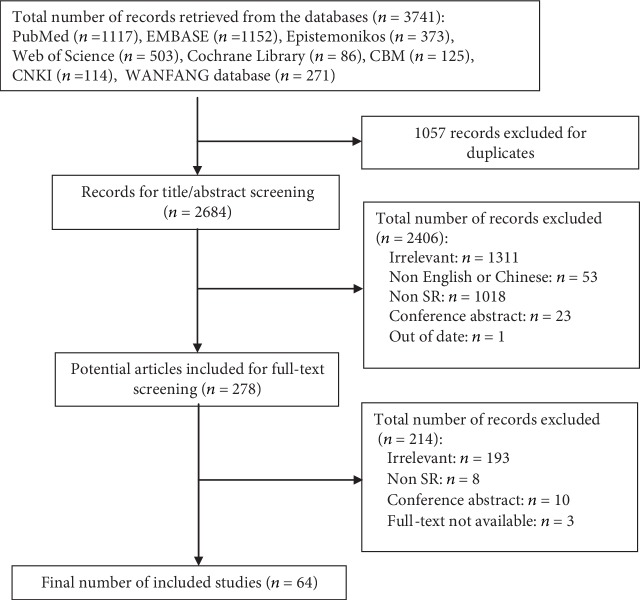
Flow chart outlining the study selection process.

A total of 3,741 literature reviews were retrieved, of which 2,684 remained after removing duplications. Following a review of titles and abstracts, 64 relevant articles were retrieved as full texts and reviewed for eligibility. A flow chart of the study selection process is shown in Figure 1.

### 3.2. Characteristics of Included Studies

Of the 64 included SRs, 15 different countries of the first author were noted. The majority of SRs (*n* = 33, 51.6%) were published in China, of which 17 were written in Chinese. The second highest number of SRs were from the United States (*n* = 5, 7.8%). For the year of publication, included SRs were published from 2004 to 2017. The largest number was published in 2015 (*n* = 15, 23.4%), followed by 2016 (*n* = 14, 21.9%). For the source of the included studies, SRs were published in 32 journals and four university degree dissertations.

Publications in Obesity Surgery were most common (*n* = 18, 28.1%) (Table 1). Of the included SRs, 5 (7.8%) reported the level of evidence, of which 4 applied the GRADE method and 1 used the Oxford grading system. Of the 64 SRs, 26 (40.6%) reported funding, and 13 (20.3%) retrieved grey literature. The median number of the authors was 6 (ranging from 1 to 14), and the median number of studies was 11 (ranging from 3 to 621). The median number of databases was 4 (ranging from 1 to 14) (see Additional [Supplementary-material supplementary-material-1]).

#### 3.2.1. AMSTAR Score

**Figure 2:**
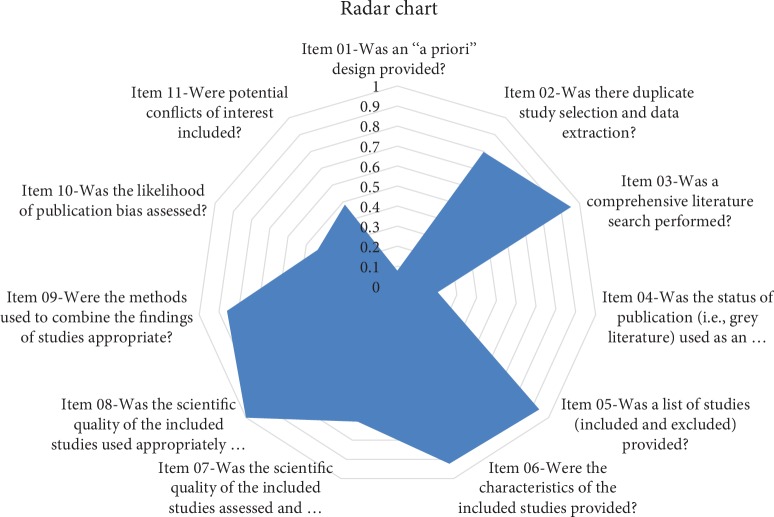
The coincidence rate for each item.

The average AMSTAR score was 7.4 ± 1.7. AMSTAR scores of 7 (*n* = 21, 32.8%) and 8 (*n* = 14, 28.1%) were most common (Table 2). There were 14 studies (21.9%) that met the criteria for high-quality SRs [28–32], of which 11 scored 9, 2 scored 10, and one scored 11. In the high-quality SRs, 11 were from China, 9 of which were published in English journals (see Additional [Supplementary-material supplementary-material-1]). Regarding the AMSTAR score for each item, the coincidence rate was over 70% and included Item 2 (79.7%), Item 3 (95.3%), Item 5 (93.8%), Item 6 (92.2%), Item 7 (70.3%), Item 8 (100%), and Item 9 (85.9%) (Table 2); the visible results are displayed in a radar chart (Figure 2).

#### 3.2.2. Publication Year

**Figure 3:**
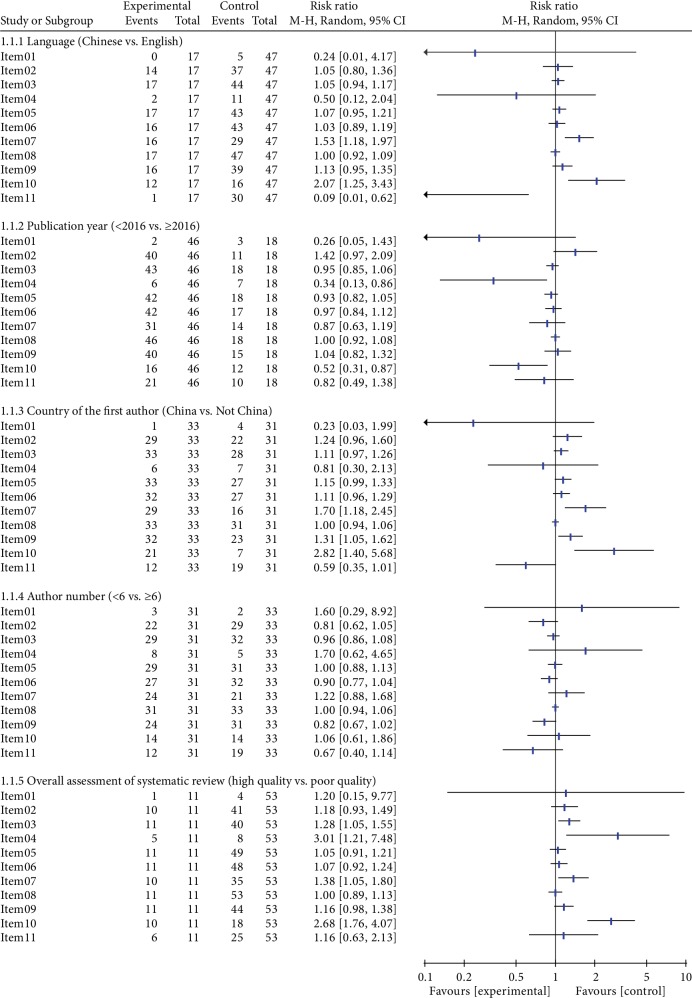
Quality assessment of each item within different subgroups (all RR (Risk Ratio) results in the figure were used a random effects model).

The AMSTAR scores of SRs published before 2016 (*n* = 46, 71.9%) compared to SRs published after 2016 (*n* = 18, 28.1%) did not significantly differ (Mean Difference (MD) = −0.79, 95% confidence interval (CI) -1.65-0.07, *P* = 0.07). Of the 11 AMSTAR items, only Item 4 (RR = 0.34, 95% CI (-1.65, 0.07)) and Item 10 (RR = 0.52, 95% CI (0.31, 0.87)) displayed significant differences (Table 3, Figure 3).

#### 3.2.3. Language

The AMSTAR scores of SRs published in Chinese (*n* = 17, 26.6%) compared to SRs published in English (*n* = 47, 73.4%) did not significantly differ (MD = 0.21, 95% CI (-0.55, 0.97), *P* = 0.59). For the 11 AMSTAR items, only Item 7 (RR = 1.53, 95% CI (1.18, 1.97)), Item 10 (RR = 2.07, 95% CI (1.25, 3.43)), and Item 11 (RR = 0.09, 95% CI (0.01, 0.62)) displayed statistically significant differences (Table 3, Figure 3).

#### 3.2.4. Country of the First Author

The AMSTAR scores of SRs published in China (*n* = 33, 51.6%) compared with SRs published outside of China (*n* = 31, 48.4%) did not significantly differ (MD = 1.10, 95% CI (0.29, 1.91), *P* = 0.008). For the 11 AMSTAR items, only Item 7 (RR = 1.70, 95% CI (1.18, 2.45)), Item 9 (RR = 1.31, 95% CI (1.05, 1.62)), and Item 10 (RR = 2.82, 95% CI (1.40, 5.68)) displayed statistically significant differences (Table 3, Figure 3).

#### 3.2.5. Author Number

The AMSTAR scores of SRs with an author number < 6 (*n* = 31, 48.4%) did not significantly differ from SRs with an author number ≥ 6 (*n* = 33, 51.6%), (MD = −0.36, 95% CI (-1.22, 0.50), *P* = 0.41). There were no statistically significant differences in the 11 AMSTAR items (Table 3, Figure 3).

#### 3.2.6. Overall Assessment of SR

The AMSTAR scores of high-quality SRs published after 2016 (*n* = 11, 17.2%) compared with other SRs (*n* = 53, 82.8%) displayed statistically significant differences (MD = 1.75, 95% CI (1.01, 2.49), *P* < 0.00001). However, for the 11 AMSTAR items, only Item 3 (RR = 1.28, 95% CI (1.05, 1.55)), Item 4 (RR = 3.01, 95% CI (1.21, 7.48)), Item 7 (RR = 1.38, 95% CI (1.05, 1.80)), and Item 10 (RR = 2.68, 95% CI (1.76, 4.07)) displayed significant differences (Table 3, Figure 3).

## 4. Discussion

This study found that the number of SRs of weight loss surgery for diabetes mellitus is increasing by year, but only a small number meet the criteria to support guideline recommendations. Subgroup analysis showed that the average AMSTAR scores of the SRs published in the last two years were higher than those of earlier years. The average scores of SRs from China were also higher than those from other countries. Specific to each item and between different subgroups (publication year, language, country of the first author, number of authors, and overall assessment of SR), there were no statistically significant differences in the results of SRs for most items.

Due to the recommendation of professional institutions and guidelines, weight loss surgery is gradually used in patients with diabetes. Simultaneously, studies on the efficacy and safety of different surgical methods recommend an increased number of SRs on the subject. However, the quantity and proportion of high-quality SRs is low; the reasons for this may include the following:

1. Most SRs lack an understanding and awareness of the protocol registration, and so the subject protocol is rarely registered or published. To benefit SR protocol registration [33], we recommend avoiding study duplication and helping healthcare workers identify differences between protocols when reporting the method or outcome, in order to confirm whether reporting bias exists. This will improve the quality of decision-making. In 2009, surveys revealed that a considerable number of SRs were not published due to the results not displaying statistical significance [34]. In 2010, the Cochrane SR found that changes to the original plan are biased and results can be misinterpreted [35]. Therefore, SR protocol registration allows transparency and addresses these concerns. To date, researchers have established six SR registration platforms, including The Cochrane Collaboration (https://www.cochranelibrary.com/), The Campbell Collaboration (https://www.campbellcollaboration.org/), The Cardiff University SR Network (https://www.caerdydd.ac.uk/insrv/libraries/sure/sysnet/), PROSPERO (https://www.crd.york.ac.uk/prospero/), The Joanna Briggs Institute registry platform (https://joannabriggs.org/), and The CAMARADES Collaboration (http://www.dcn.ed.ac.uk/camarades/default.htm) that involve intervention, diagnosis, prognosis, and methodology. These can be applied to fields that involve clinical, society, psychology, education, and criminal justice and criminology, in addition to SRs of different categories such as human and animal studies
2. The majority of SRs did not retrieve the grey literature, which increased the risk of leak detection and thus affected any estimations of the effect
3. Most of the SRs considered publication type for eligibility (such as meeting abstracts). Studies have shown that approximately 10% of the references in the Cochrane SR are meeting abstracts or other grey literature [36]. It has been shown that for studies of the same subject, the efficacy of published trials is higher than that reported in the grey literature [37], and thus, the accuracy of SR results can be affected by unretrieved and excluded grey literature
4. The majority of SRs possessed undetected publication bias. Acquiring a small number of early SRs would overestimate the effects, particularly when negative results are published [38]. It is therefore of great importance to evaluate the accuracy of publication bias to determine the results of the SR
5. The majority of SR participants did not declare conflicts of interest, which may lead to biased conclusions [39, 40]. Stating that no conflicts of interest exist is conducive to the high-quality decision of the evidence and for the application of the results to health policy makers
6. Researchers had insufficient knowledge of the grading method, or the GRADE method was used incorrectly. In the included SRs, 5 articles conducted evidence classification [18–22], and 4 applied the GRADE method [18–20, 22], but 2 studies used this incorrectly [18, 19]. An SR of the downgrade factors must be considered when grading the evidence, including study quality, generalizability, and heterogeneity [18]. The level of evidence also did not follow high, moderate, low, and very low classifications. Another SR sequentially used GRADE on individual studies and failed to understand the concept of the evidence [19]. The use of the GRADE method in SRs can help the authors interpret the results and can be beneficial to the readers' understanding, but its use can also be inaccurate or misleading to readers

The strengths of this study include the following: (1) the systematic and comprehensive retrieval and collection of SRs of bariatric surgery in diabetic patients, (2) the systematic evaluation of the quality of the SR to provide references for guideline developers and policy makers, and (3) the AMSTAR scores of different subgroups being visualized using forest plots to allow the reader to intuitively understand the quality of the SR. The study was limited by the fact that we only focused on the methodological quality of SRs and did not use the GRADE method to interpret the evidence of SRs.

In order to strengthen the methodological quality of bariatric surgery systematic reviews, we made the following specific recommendations:

1. Write a detailed study protocol outlining end points, inclusion criteria, and a search strategy, and publish it in advance on a publically available website (e.g., PROSPERO)
2. Report the study in such a way as to allow reproducibility of the results (PRISMA) or future updating of the systematic review
3. Include an experienced meta-analyst, content expert (ideally, a triallist), and statistician

Be circumspect when interpreting the results; acknowledge the sources of bias; and consider heterogeneity, generalizability, and contemporary clinical relevance.

## 5. Conclusion

The number of SRs assessing the efficacy of bariatric surgery in diabetic patients is increasing on a yearly basis, but only a small number of SRs meet the criteria to support guideline recommendations. Study protocols not being registered, grey literature not retrieved, incorporation of grey literature as exclusion criteria, a lack of evaluation of publication bias, and failing to report conflicts of interest are the major causes of low AMSTAR scores.

## Figures and Tables

**Table 1:** Top ten of countries, year of publication, and literature resources included in the SR.

Country of the first author	Number	Publication year	Number	Literature resource	Number
China	33	2015	15	Obesity Surgery	18
USA	5	2016	14	Surgery for Obesity and Related Diseases	6
Canada	4	2014	9	Chinese Journal of Evidence-Based Medicine	4
Germany	3	2013	9	Academic dissertation	4
Korea	3	2012	6	Annals of Surgery	2
Australia	3	2017	4	Diabetes/Metabolism Research and Reviews	2
UK	3	2011	2	Obesity Reviews	2
Italy	2	2010	2	JAMA	1
Netherlands	2	2009	1	Diabetes, Obesity and Metabolism	1
Others^#^	6	Others^$^	2	Others^&^	24

^#^Each of the following countries published one: Brazil, Czech Republic, France, New Zealand, Singapore, and Switzerland; ^$^one in 2004 and one in 2007; ^&^involved 24 journals.

**Table 2:** AMSTAR scores (number/proportion).

Item	Yes	No	Cannot answer	Not applicable
Item 1	5/7.8%	59/92.2%	0/0	0/0
Item 2	51/79.7%	0/0	13/20.3%	0/0
Item 3	61/95.3%	3/4.7%	0/0	0/0
Item 4	13/20.3%	51/79.7%	0/0	0/0
Item 5	60/93.8%	4/6.2%	0/0	0/0
Item 6	59/92.2%	5/7.8%	0/0	0/0
Item 7	45/70.3%	0/0	19/29.7%	0/0
Item 8	64/100%	0/0	0/0	0/0
Item 9	55/85.9%	0/0	0/0	9/14.1%
Item 10	28/43.8%	0/0	36/56.2%	0/0
Item 11	31/48.4%	33/51.6%	0/0	0/0

**Table 3:** SRs of AMSTAR scores in different subgroups.

Subgroup	Number	Mean ± SD	*P* value
*Publication year*			
<2016	46	7.1 ± 1.8	0.07
≥2016	18	7.9 ± 1.5	
*Language*			
Chinese	17	7.5 ± 1.1	0.59
English	47	7.3 ± 1.9	
*Country of the first author*			
China	33	7.9 ± 1.2	0.008
Not China	31	6.8 ± 1.9	
*The number of author*			
<6 authors	31	7.2 ± 2.2	0.41
≥6 authors	33	7.6 ± 1.2	
*Overall assessment of SR*			
High quality^∗^	11	8.8 ± 1.0	<0.00001
Poor quality	53	7.1 ± 1.7	

^∗^AMSTAR score ≥ 9 and publication year ≥ 2016.

## Data Availability

The data used to support the findings of this study are available from the corresponding authors upon request.

## Supplementary Materials

Supplementary 1 Additional file 1: search strategy.

Supplementary 2 Additional file 2: AMSTAR Tool.

Supplementary 3 Additional file 3: characteristics of included studies.

Supplementary 4 Additional file 4: quality assessment of each study.
