# Supplementary material for: Quality Assessment of Systematic Review of the Bariatric Surgery for Diabetes Mellitus
Source: J Diabetes Res. 2019 Nov 21;2019:9541638. doi: 10.1155/2019/9541638 (PMC6906849; doi:10.1155/2019/9541638)
Supplement: Supplementary 1 — Additional file 1: search strategy. [file 9541638.f1.pdf]

Additional file 1：Search strategy

Medline（N=1117）

#1 "Perioperative Period"[Mesh]
#2 "Perioperative Care"[Mesh]
#3 "Surgical Procedures, Operative"[Mesh]
#4 perioperative[Title/Abstract]
#5 "peri-operative"[Title/Abstract]
#6 preoperative[Title/Abstract]
#7 "pre-operative"[Title/Abstract]
#8 postoperative[Title/Abstract]
#9 "post-operative"[Title/Abstract]
#10 "pre-surgery"[Title/Abstract]
#11 "peri-surgery"[Title/Abstract]
#12 "post-surgery"[Title/Abstract]
#13 intraoperative[Title/Abstract]
#14 "intra-operative"[Title/Abstract]
#15 surgical[Title/Abstract]
#16 OR/#1-15
#17 "Diabetes Mellitus"[Mesh]
#18 diabet*[Title/Abstract]
#19 IDDM[Title/Abstract]
#20 NIDDM[Title/Abstract]
#21 MODY[Title/Abstract]
#22 T2DM[Title/Abstract]
#23 T2D[Title/Abstract]
#24 T1DM[Title/Abstract]
#25 T1D[Title/Abstract]
#26 OR/#17-25
#27 "Meta-Analysis" [Publication Type]
#28 "Meta-Analysis as Topic"[Mesh]
#29 "systematic review"[Title/Abstract]
#30 "meta analysis"[Title/Abstract]
#31 "meta-analysis"[Title/Abstract]
#32 "meta analyses"[Title/Abstract]
#33 "meta-analyses"[Title/Abstract]
#34 OR/#27-33
#35 #16 AND #26 AND #34

EMBASE（N=1152）

#1 'perioperative period'/exp
#2 'perioperative nursing'/exp
#3 'surgery'/exp
#4 perioperative:ab,ti

#5 ‘peri-operative’:ab,ti
#6 preoperative:ab,ti
#7 ‘pre-operative’:ab,ti
#8 postoperative:ab,ti
#9 ‘post-operative’:ab,ti
#10 ‘pre-surgery’:ab,ti
#11 ‘peri-surgery’:ab,ti
#12 ‘post-surgery’:ab,ti
#13 intraoperative:ab,ti
#14 ‘intra-operative’:ab,ti
#15 surgical:ab,ti
#16 OR/#1-15
#17 'diabetes mellitus'/exp
#18 diabet*:ab,ti
#19 IDDM:ab,ti
#20 NIDDM:ab,ti
#21 MODY:ab,ti
#22 T2DM:ab,ti
#23 T2D:ab,ti
#24 T1DM:ab,ti
#25 T1D:ab,ti
#26 OR/#17-25
#27 'meta analysis'/exp
#28 ‘systematic review’:ab,ti
#29 ‘meta analysis’:ab,ti
#30 ‘meta-analysis’:ab,ti
#31 ‘meta analyses’:ab,ti
#32 ‘meta-analyses’:ab,ti
#33 OR/#27-32
#34 #16 AND #26 AND #33
#35 #34 AND [medline]/lim
#36 #34 NOT #35

Epistemonikos（N=373）

#1 perioperative[Title/Abstract]
#2 "peri-operative" [Title/Abstract]
#3 preoperative[Title/Abstract]
#4 "pre-operative"[Title/Abstract]
#5 postoperative[Title/Abstract]
#6 "post-operative"[Title/Abstract]
#7 "pre-surgery"[Title/Abstract]
#8 "peri-surgery"[Title/Abstract]
#9 "post-surgery"[Title/Abstract]
#10 intraoperative[Title/Abstract]

#11 "intra-operative"[Title/Abstract]
#12 surgical[Title/Abstract]
#13 OR/#1-12
#14 diabet*[Title/Abstract]
#15 IDDM[Title/Abstract]
#16 NIDDM[Title/Abstract]
#17 MODY[Title/Abstract]
#18 T2DM[Title/Abstract]
#19 T2D[Title/Abstract]
#20 T1DM[Title/Abstract]
#21 T1D[Title/Abstract]
#22 OR/#14-21
#23 #13 AND #22
#24 #23 AND limit [systematic review]

Web of Science（N=503）

#1 TOPIC:perioperative
#2 TOPIC:"peri-operative"
#3 TOPIC:preoperative
#4 TOPIC:"pre-operative"
#5 TOPIC:postoperative
#6 TOPIC:"post-operative"
#7 TOPIC:"pre-surgery"
#8 TOPIC:"peri-surgery"
#9 TOPIC:"post-surgery"
#10 TOPIC:intraoperative
#11 TOPIC:"intra-operative"
#12 TOPIC:surgical
#13 OR/#1-12
#14 TOPIC:diabet*
#15 TOPIC:IDDM
#16 TOPIC:NIDDM
#17 TOPIC:MODY
#18 TOPIC:T2DM
#19 TOPIC:T2D
#20 TOPIC:T1DM
#21 TOPIC:T1D
#22 OR/#14-21
#23 TOPIC:"systematic review"
#24 TOPIC:"meta analysis"
#25 TOPIC:"meta-analysis"
#26 TOPIC:"meta analyses"
#27 TOPIC:"meta-analyses"
#28 OR/#23-27

#30 #13 AND #22 AND #28

Cochrane Library（N=86）

#1 MeSH descriptor: [Surgical Procedures, Operative] explode all trees
#2 perioperative:ti,ab,kw
#3 "peri-operative":ti,ab,kw
#4 preoperative:ti,ab,kw
#5 "pre-operative":ti,ab,kw
#6 postoperative:ti,ab,kw
#7 "post-operative":ti,ab,kw
#8 "pre-surgery":ti,ab,kw
#9 "peri-surgery":ti,ab,kw
#10 "post-surgery":ti,ab,kw
#11 intraoperative:ti,ab,kw
#12 "intra-operative":ti,ab,kw
#13 surgical:ti,ab,kw
#14 OR/#1-13
#15 MeSH descriptor: [Diabetes Mellitus] explode all trees
#16 diabet*:ti,ab,kw
#17 IDDM:ti,ab,kw
#18 NIDDM:ti,ab,kw
#19 MODY:ti,ab,kw
#20 T2DM:ti,ab,kw
#21 T2D:ti,ab,kw
#22 T1DM:ti,ab,kw
#23 T1D:ti,ab,kw
#24 OR/#15-23
#25 #14 AND #24
#26 #25 AND limit [Cochrane Review]

CBM（N=125）

#1 主题词:手术期间/全部树/全部副主题词
#2 主题词:手术中并发症/全部树/全部副主题词
#3 主题词:围手术期护理/全部树/全部副主题词
#4 主题词:围手术期医护/全部树/全部副主题词
#5 缺省[智能]:手术
#6 缺省[智能]:围术期
#7 缺省[智能]:术前
#8 缺省[智能]:术中
#9 缺省[智能]:术后
#10 or/#1-9
#11 主题词:糖尿病/全部树/全部副主题词
#12 缺省[智能]:糖尿病
#13 or/#11-12

#14 主题词:Meta 分析/全部树/全部副主题词
#15 主题词:Meta 分析(主题)/全部树/全部副主题词
#16 缺省[智能]:meta
#17 缺省[智能]:系统评价
#18 缺省[智能]:系统综述
#19 缺省[智能]:荟萃分析
#20 or/#14-19
#21 #10 and #13 and #20

CNKI（N=114）
#1 主题=手术
#2 主题=围术期
#3 主题=术前
#4 主题=术中
#5 主题=术后
#6 or/#1-5
#7 主题=糖尿病
#8 主题=meta
#9 主题=系统评价
#10 主题=系统综述
#11 主题=荟萃分析
#12 or/#8-11
#13 #6 and #7 and #12

万方（N=271）
#1 主题:手术
#2 主题:围术期
#3 主题:术前
#4 主题:术中
#5 主题:术后
#6 or/#1-5
#7 主题:糖尿病
#8 主题: meta
#9 主题:系统评价
#10 主题:系统综述
#11 主题:荟萃分析
#12 or/#8-11
#13 #6 and #7 and #12
