# Supplementary material for: Quality Assessment of Systematic Review of the Bariatric Surgery for Diabetes Mellitus
Source: J Diabetes Res. 2019 Nov 21;2019:9541638. doi: 10.1155/2019/9541638 (PMC6906849; doi:10.1155/2019/9541638)
Supplement: Supplementary 3 — Additional file 3: characteristics of included studies. [file 9541638.f3.pdf]

Additional file 3: Characteristics of included studies

Title	Publication year	Journal	language	No. of authors	Country	Style of included studies	No. of included studies	Simple size
Obesity, bariatric surgery and type 2 diabetes – a systematic review	2004	Diabetes/Metabolism Research and Reviews	English	2	USA	RCT	--*	--*
The Comparative Effects of Bariatric Surgery on Weight and Type 2 Diabetes	2007	Obesity Surgery	English	4	France	2 meta-analyses, 21 primary studies	23	--**
Weight and Type 2 Diabetes after Bariatric Surgery: Systematic Review and Meta-analysis	2009	The American Journal of Medicine	English	8	USA	29 RCTs, 49 nRCT PSs, 60 comparative RSs, 187 uncontrolled CSs PSs, 266 single-arm RSs, 25 OSs, 2 CCSs	621	135246
Metabolic Surgery for the Treatment of Type 2 Diabetes in Patients with BMI <35 kg/m2: An Integrative Review of Early Studies	2010	Obesity Surgery	English	6	Czech Republic	prospective OS	16	343
Sleeve gastrectomy and type 2 diabetes mellitus: a systematic review	2010	Surgery for Obesity and Related Diseases	English	5	Canada	3 prospective nonRCTs, 3 retrospective controlled trials,	27	673

						15 prospective CSs, 6 retrospective CSs		
Bariatric Surgery as a Novel Treatment for Type 2 Diabetes Mellitus：A Systematic Review	2011	Archives of Surgery	English	6	Netherlands	1 RCT, 8 OSs	9	82988
Effectiveness of surgical weight loss on the remission of type 2 diabetes mellitus: A systematic review	2011	JBI Library of systematic review	English	2	Australia	RCT	5	not report
Ethnic Differences in Weight Loss and Diabetes Remission After Bariatric Surgery： A meta-analysis	2012	Diabetes Care	English	6	Netherlands	9 prospective OSs, 5 ROSs	14	3801
Impact of laparoscopic adjustable gastric banding on type 2 diabetes	2012	Obesity Reviews	English	4	Australia	23 CSs, 4 nonrandomized CCSs, 6 RCTs, 2 retrospective data analysis	35	1264
Metabolic effects of bariatric surgery in type 2 diabetic patients with body mass index < 35 kg/m2	2012	Diabetes, Obesity and Metabolism	English	14	China	2 RSs, 11 PSs	13	357

[A SYSTEMATIC REVIEW AND META-ANALYSIS 0F BARIATRIC SURGERY oN THE TREATMENT oF TYPE 2 DIABETES MELLITUS]	2012	Southeast University -academic dissertation	China	1	China	RCT；nonRCT；retrospective case serie；prospective case series	28	695
Laparoscopic sleeve gastrectomy and Roux-en-Y gastro-intestinal bypass for the treatment of type 2 diabetes: a Meta-analysis of prospective research	2012	LINGNAN MODERN CLINICS IN SURGERY	China	4	China	2 RCTs, 4 prospective cohort studies	6	464
[One-Year Postoperative Effects of Gastric Bypass on Type 2 Diabetes in Mainland China: A Meta-Analysis]	2012	Chinese Journal of Evidence-Based Medicine	China	6	China	BAS	6	131
Bariatric Surgery for Weight Loss and Glycemic Control in Nonmorbidly Obese Adults With Diabetes： A Systematic Review	2013	JAMA	English	8	USA	RCT	3	290
Comparison of laparoscopic Roux-en-Y gastric bypass with laparoscopic sleeve gastrectomy for morbid obesity or type 2 diabetes mellitus: a meta-analysis of randomized controlled trials	2013	The Journal of the Canadian Chiropractic	English	4	China	RCTs	5	396
Gastric Bypass and Sleeve Gastrectomy for Type 2 Diabetes:A Systematic Review and Meta-analysis of Outcomes	2013	Obesity Surgery	English	3	New Zealand	21 PSs (3 RCTs), 12 RSs	33	1375
Role of Bariatric Surgery as Treatment for Type 2 Diabetes in Patients Who Do Not Meet Current NIH Criteria: A Systematic Review and Meta-Analysis	2013	Journal of the American College of Surgeons	English	9	USA	22 PSs（3 RCTs）, 17 RSs	39	1389

The Effects of Bariatric Procedures versus Medical Therapy for Obese Patients with Type 2 Diabetes: Meta-Analysis of Randomized Controlled Trials	2013	BioMed Research International	English	6	China	RCT	3	270
[Bariatric Surgery versus Conventional Medical Therapy for Obese Patients with Type 2 Diabetes: A Meta-Analysis]	2013	Chinese Journal of Evidence-Based Medicine	China	5	China	RCT	3	340
[Efficacies of sleeve gastrectomy and Roux-en-Y gastric bypass for the treatment of type 2 diabetes mellitus: a Meta-analysis]	2013	Chinese Journal of Digestive Surgery	China	6	China	RCT	5	348
[Bariatric surgery for non-obese type 2 diabetes mellitus in Mainland China:A meta-analysis]	2013	Journal of Clinical Rehabilitative Tissue Engineering Research	China	9	China	BAS	7	307
[Bariatric Surgery for Obese Type 2 Diabetes Patients in Mainland China: A Systematic Review]	2013	Chinese Journal of Evidence-Based Medicine	China	7	China	BAS	6	100
[Hypoglycemic effects of three types of gastrointestinal reconstruction on type 2 diabetes mellitus : a meta-analysis]	2014	Medical Journal of the Chinese People's Armed Police Forces	China	5	China	1 RCT, 1 quasi RCT, 5 nonRCTs	7	378
A Meta-analysis of 2-Year Effect After Surgery:Laparoscopic Roux-en-Y Gastric Bypass Versus Laparoscopic Sleeve Gastrectomy for Morbid Obesity and Diabetes Mellitus	2014	Obesity Surgery	English	4	China	1 RCT, 11 PCTs, 4 ROSs	16	9756
Correlation between Postoperative Weight Loss and Diabetes Mellitus Remission: A	2014	Obesity Surgery	English	4	China	RCTs	8	1247

Meta-Analysis								
Diabetes and Weight in Comparative Studies of Bariatric Surgery vs Conventional Medical Therapy: A Systematic Review and Meta-Analysis	2014	Obesity Surgery	English	3	Germany	5 RCTs, 11 nonRCTs	16	6131
Early Impact of Bariatric Surgery on Type II Diabetes,Hypertension, and Hyperlipidemia: A Systematic Review,Meta-Analysis and Meta-Regression on 6,587 Patients	2014	Obesity Surgery	English	5	Germany	RCT	19	6587
Efficacy of Metabolic Surgery on HbA1c Decrease in Type 2 Diabetes Mellitus Patients with BMI <35 kg/m2—a Review	2014	Obesity Surgery	English	4	Singapore	4 RCTs, 36 prospective observational or uncontrolled studies, 13 RSs	53	2258
Impact of roux-en Y gastric bypass surgery on prognostic factors of type 2 diabetes mellitus: meta-analysis and systematic review	2014	Diabetes/Metabolism Research and Reviews	English	6	China	not report	10	924
The Duodenal–Jejunal Bypass Liner for the Treatment of Type 2 Diabetes Mellitus and/or Obesity: a Systematic Review	2014	Obesity Surgery	English	3	Austria	6 CSs, 3 RCTs, 1 non-RCT	10	342
[Short- and long-term remission of obese type 2 diabetes mellitus after gastric bypass surgery or sleeve gastrectomy： a Meta analysis]	2014	Chinese Archives of General Surgery (Electronic Edition)	China	4	China	RCT	34	2490
A Meta-Analysis of Short-Term Outcomes of Patients with Type 2 Diabetes Mellitus and BMI	2015	World Journal of Surgery	English	5	China	PS	9	343

£35 kg/m2 Undergoing Roux-en-Y Gastric Bypass								
A systematic review and meta-analysis of the effect of Billroth reconstruction on type 2 diabetes: A new perspective on old surgical methods	2015	Surgery for Obesity and Related Diseases	English	6	Korea	RCS	8	972
Bariatric Surgery for Type 2 Diabetes Mellitus in Patients with BMI <30 kg/m2: A Systematic Review and Meta-Analysis	2015	PLOS ONE	English	5	China	PS	10	290
Effect of sleeve gastrectomy on type 2 diabetes as an alternative treatment modality to Roux-en-Y gastric bypass: systemic review and meta-analysis	2015	Surgery for Obesity and Related Diseases	English	6	Korea	3 retrospective clinical studies, 6 PCSs, 2 RCTs	11	857
Efficacy of Laparoscopic Mini Gastric Bypass for Obesity and Type 2 Diabetes Mellitus: A Systematic Review and Meta-Analysis	2015	Gastroenterology Research and Practice	English	8	China	RCT	31	17061
Laparoscopic Roux-en-Y Gastric Bypass versus Sleeve Gastrectomy for Obese Patients with Type 2 Diabetes: A Meta-analysis of Randomized Controlled Trials	2015	The American Journal of Surgery	English	6	China	RCT	4	256
Long-Term Effects of Bariatric Surgery on Type II Diabetes,Hypertension and Hyperlipidemia: A Meta-Analysis and Meta-Regression Study with 5-Year Follow-Up	2015	Obesity Surgery	English	6	Germany	prospective cohort study	22	4160
Predictive Factors of Type 2 Diabetes Mellitus Remission Following Bariatric Surgery: a	2015	Obesity Surgery	English	11	China	9 RSs, 5 PSs, 1 OS	15	1753

Meta-analysis								
Predictors of Remission of Diabetes Mellitus in Severely Obese Individuals Undergoing Bariatric Surgery: Do BMI or Procedure Choice Matter? A Meta-analysis	2015	Annals of Surgery	English	4	Italy	6 RCTs, 51 nonRCTs, 4 nonRCTs, 4 controlled OSs, 26 non controlled OSs, 2 controlled studies, 1 comprehensive study	94	94579
Surgical Versus Medical Treatment of Type 2 Diabetes Mellitus in Nonseverely Obese Patients：A Systematic Review and Meta-analysis	2015	Annals of Surgery	English	11	Switzerland	6 observational clinical studies, 5 RCTs	11	706
The Impact of Bariatric Surgery on Diabetic Retinopathy:A Systematic Review and Meta-Analysis	2015	Obesity Surgery	English	6	Canada	non-randomized CS	4	148
The Long-Term Effects of Bariatric Surgery for Type 2 Diabetes:Systematic Review and Meta-analysis of Randomized and Non-randomized Evidence	2015	Obesity Surgery	English	7	China	2 RCTs, 24 cohort studies	26	7883
Type 1 Diabetes Mellitus and Bariatric Surgery: A Systematic Review and Meta-Analysis	2015	Obesity Surgery	English	8	UK	6 CPs, 22 CSs	28	148
[Laparoscopic Roux-en-Y Gastric Bypass versus Laparoscopic Sleeve Gastrectomy for Obesity and	2015	Chinese Journal of Evidence-Based	China	7	China	RCT	8	828

Type 2 Diabetes Mellitus: A Meta-analysis]		Medicine						
[Short-Term Efficacy of Laparoscopic Sleeve Gastrectomy for Obesity with Type 2 Diabetes Mellitus in China: A Systematic Analysis]	2015	Chinese Journal of Bases and Clinics in General Surgery	China	4	China	CSs	7	107
Bariatric Surgery in Type 1 Diabetes Mellitus: A Systematic Review	2016	Obesity Surgery	English	6	UK	3 retrospective Comparative studies, 8 CSs, 4 CPs	15	68
[A META-ANALYSIS OF SLEEVE GASTRECTOMY VERSUS ROUX-EN-Y GASTRIC BYPASS FOR MORBID OBESITY AND DIABETES MELLITUS]	2016	Journal of Inner Mongolia Medical University	China	7	China	RCT	9	713
[Three types of gastrointestinal reconstruction on non-obese type 2 diabetes mellitus: a meta-analysis]	2017	Anhui Medical Journal	China	3	China	observational studies（8）	20	956
A Systematic Review and Meta-Analysis of Outcomes for Type 1 Diabetes after Bariatric Surgery	2016	Journal of Obesity	English	8	Canada	11 CSs , 2CPs	13	86
Bariatric surgery and diabetic retinopathy_ a systematic review and meta-analysis of controlled clinical studies	2017	obesity reviews	English	4	Italy	retrospective cohort study、randomized controlled tria、case–control study	7	16651

Bariatric surgery is associated with less progression of diabetic retinopathyA systematic review and metaanalysis	2016	Surgery for Obesity and Related Diseases	English	6	Korea	six retrospective observational case series, two retrospective case-control studies, two prospective case-control studies, and two randomized controlled trials	12	876
Bariatric surgery reduces urinary albumin excretion in diabetic nephropathy_ a systematic review and meta-analysis	2016	Surgery for Obesity and Related Diseases	English	5	USA		15	1839
Impact of bariatric surgery on renal functions in patients with type 2 diabetes mellitus systematic review of randomized trials and observational studies	2016	Surgery for Obesity and Related Diseases	English	6	China	4 RCTs, 5 cohort studies, 20 BASs	29	18172
Meta-analysis of bariatric surgery versus non-surgical treatment for type 2 diabetes mellitus	2016	Oncotarget	English	8	China	RCT	8	619
Preoperative Fasting Plasma C-Peptide Levels as Predictors of Remission of Type 2 Diabetes Mellitus after Bariatric Surgery: A Systematic Review and Meta-Analysis	2016	Journal of Investigative Surgery	English	4	China	6PS、 8ROS	14	991
Roux-En-Y Gastric Bypass in Type 2 Diabetes	2017	Obesity Surgery	English	5	Brazil	RCT	5	342

Patients with Mild Obesity_ a Systematic Review and Meta-analysis								
Roux-en-Y Gastric Bypass Versus Medical Treatment for Type 2 Diabetes Mellitus in Obese Patients_ A Systematic Review and Meta-Analysis of Randomized Controlled Trials	2016	Medicine	English	10	China	RCT	6	410
Sleeve Gastrectomy and Type 2 Diabetes Mellitus_ a Systematic Review of Long-Term Outcomes	2016	Obesity Surgery	English	6	Canada	10CSs, with 1RCT.	11	1354
Type 1 Diabetes Mellitus and Bariatric Surgery_ A Systematic Review and Meta-Analysis	2016	Obesity Surgery	English	8	UK	CS ,CP	28	142
[Meta-analysis of traditional treatment and weight loss surgery in the clinical treatment of obese type 2 diabetes patients]	2016	Yiayao Qianyan	China	2	China	not mentionrd	30	--*
[A Meta-analysis of therapeutic effect and related complications of laparoscopic sleeve gastrectomy and laparaoscopic Roux-en-Y gastric bypass]	2016	Academic dissertation-Lanzhou University	China	1	China	RCT	15	4037
[The Short-Term Effect Analysis of Laparoscopic Sleeve Gastrectomy and Laparoscopic gastric bypass for obesity and type 2 diabetes（T2DM） in China]	2016	Academic dissertation- Hebei Medical University	China	1	China	RCT	10	--*
[Comparative on clinical effect and cost-effectiveness Bariatric Surger for patients with type 2 Diabetes Mellitus]	2017	Academic dissertation- Shandong University	China	1	China	3RCT、2RCS	5	1087

RCT: randomized controlled trial, PCT: parallel controlled trial, ROS: retrospective observational study, PS: prospective study, RCS: retrospective cohort study, OS: observational study, CS: case serie, CP: case report, PCS: prospective clinical study, RS: retrospective study, CCS: case-control study, BAS: before-after study in the same patient.

＊: The SR described the results qualitatively, which did not report this data. ＊＊: This study was a comprehensive review which included meta-analysis and original study as they were relevant to the topic. In this review, the results were described qualitatively, which did not report this data.
