# Supplementary material for: Quality Assessment of Systematic Review of the Bariatric Surgery for Diabetes Mellitus
Source: J Diabetes Res. 2019 Nov 21;2019:9541638. doi: 10.1155/2019/9541638 (PMC6906849; doi:10.1155/2019/9541638)
Supplement: Supplementary 4 — Additional file 4: quality assessment of each study. [file 9541638.f4.pdf]

Additional file 4: quality assessment of each study

No	Title	Item 01-Was an ‘‘a priori’’ design provided?	Item 02-Was there duplicate study selection and data extraction?	Item 03-Was a comprehensive literature search performed?	Item 04-Was the status of publication (i.e., grey literature) used as an inclusion criterion?	Item 05-Was a list of studies (included and excluded) provided?	Item 06-Were the characteristics of the included studies provided?	Item 07-Was the scientific quality of the included studies assessed and documented?	Item 08-Was the scientific quality of the included studies used appropriately in formulating conclusions?	Item 09-Were the methods used to combine the findings of studies appropriate?	Item 10-Was the likelihood of publication bias assessed?	Item 11-Were potential conflicts of interest included?	Total
1	Obesity, bariatric surgery and type 2 diabetes – a systematic review	no	can't answer	no	no	no	no	can't answer	yes	can't answer	can't answer	no	1
2	The Comparative Effects of Bariatric Surgery on Weight and Type 2 Diabetes	no	can't answer	no	no	no	no	can't answer	yes	can't answer	can't answer	no	1
3	Weight and Type 2 Diabetes after Bariatric Surgery: Systematic Review and Meta-analysis	no	yes	yes	no	no	yes	yes	yes	yes	can't answer	yes	7
4	Metabolic Surgery for the Treatment of Type 2 Diabetes in Patients with BMI <35 kg/m2: An Integrative Review of Early Studies	no	yes	yes	no	yes	yes	can't answer	yes	yes	can't answer	no	6

5	Sleeve gastrectomy and type 2 diabetes mellitus: a systematic review	no	yes	yes	yes	yes	yes	can't answer	yes	can't answer	can't answer	yes	7
6	Bariatric Surgery as a Novel Treatment for Type 2 Diabetes Mellitus： A Systematic Review	no	yes	no	no	yes	yes	can't answer	yes	can't answer	can't answer	yes	5
7	Effectiveness of surgical weight loss on the remission of type 2 diabetes mellitus: A systematic review	no	yes	yes	yes	yes	no	yes	yes	can't answer	can't answer	no	6
8	Ethnic Differences in Weight Loss and Diabetes Remission After Bariatric Surgery： A meta-analysis	no	yes	yes	no	yes	yes	yes	yes	yes	yes	yes	9
9	Impact of laparoscopic adjustable gastric banding on type 2 diabetes	no	yes	yes	no	yes	yes	yes	yes	yes	can't answer	no	7
10	Metabolic effects of bariatric surgery in type 2 diabetic patients with body mass index < 35 kg/m2	no	yes	yes	no	yes	yes	can't answer	yes	yes	can't answer	yes	7
11	[A SYSTEMATIC REVIEW AND META-ANALYSIS 0F BARIATRIC SURGERY oN THE TREATMENT oF TYPE 2 DIABETES MELLITUS]	no	yes	yes	no	yes	yes	yes	yes	yes	yes	no	8
12	Laparoscopic sleeve gastrectomy and Roux-en-Y gastro-intestinal bypass for the treatment of type 2 diabetes: a Meta-analysis of prospective research	no	yes	yes	no	yes	yes	yes	yes	yes	can't answer	no	7

13	[One-Year Postoperative Effects of Gastric Bypass on Type 2 Diabetes in Mainland China: A Meta-Analysis]	no	yes	yes	no	yes	yes	yes	yes	yes	yes	no	8
14	Bariatric Surgery for Weight Loss and Glycemic Control in Nonmorbidly Obese Adults With Diabetes： A Systematic Review	yes	yes	yes	no	yes	yes	can't answer	yes	yes	can't answer	yes	8
15	Comparison of laparoscopic Roux-en-Y gastric bypass with laparoscopic sleeve gastrectomy for morbid obesity or type 2 diabetes mellitus: a meta-analysis of randomized controlled trials	no	yes	yes	no	yes	yes	yes	yes	yes	can't answer	no	7
16	Gastric Bypass and Sleeve Gastrectomy for Type 2 Diabetes:A Systematic Review and Meta-analysis of Outcomes	no	can't answer	yes	no	yes	yes	yes	yes	yes	can't answer	no	6
17	Role of Bariatric Surgery as Treatment for Type 2 Diabetes in Patients Who Do Not Meet Current NIH Criteria: A Systematic Review and Meta-Analysis	no	can't answer	yes	no	no	yes	can't answer	yes	yes	can't answer	yes	5
18	The Effects of Bariatric Procedures versus Medical Therapy for Obese Patients with Type 2 Diabetes: Meta-Analysis of Randomized Controlled Trials	no	yes	yes	no	yes	yes	yes	yes	yes	can't answer	yes	8

19	[Bariatric Surgery versus Conventional Medical Therapy for Obese Patients with Type 2 Diabetes: A Meta-Analysis]	no	yes	yes	no	yes	yes	yes	yes	yes	can't answer	no	7
20	[Efficacies of sleeve gastrectomy and Roux-en-Y gastric bypass for the treatment of type 2 diabetes mellitus: a Meta-analysis]	no	yes	yes	no	yes	yes	yes	yes	yes	can't answer	no	7
21	[Bariatric surgery for non-obese type 2 diabetes mellitus in Mainland China:A meta-analysis]	no	yes	yes	no	yes	yes	yes	yes	yes	yes	yes	9
22	[Bariatric Surgery for Obese Type 2 Diabetes Patients in Mainland China: A Systematic Review]	no	yes	yes	no	yes	yes	yes	yes	yes	yes	no	8
23	[Hypoglycemic effects of three types of gastrointestinal reconstruction on type 2 diabetes mellitus : a meta-analysis]	no	yes	yes	yes	yes	yes	can't answer	yes	yes	yes	no	8
24	A Meta-analysis of 2-Year Effect After Surgery:Laparoscopic Roux-en-Y Gastric Bypass Versus Laparoscopic Sleeve Gastrectomy for Morbid Obesity and Diabetes Mellitus	no	yes	yes	no	yes	yes	yes	yes	yes	yes	yes	9
25	Correlation between Postoperative Weight Loss and Diabetes Mellitus Remission: A Meta-Analysis	no	yes	yes	no	yes	yes	yes	yes	yes	yes	yes	9

26	Diabetes and Weight in Comparative Studies of Bariatric Surgery vs Conventional Medical Therapy: A Systematic Review and Meta-Analysis	no	yes	yes	no	yes	yes	yes	yes	yes	can't answer	yes	8
27	Early Impact of Bariatric Surgery on Type II Diabetes,Hypertension, and Hyperlipidemia: A Systematic Review,Meta-Analysis and Meta-Regression on 6,587 Patients	no	yes	yes	no	yes	yes	can't answer	yes	yes	yes	no	7
28	Efficacy of Metabolic Surgery on HbA1c Decrease in Type 2 Diabetes Mellitus Patients with BMI <35 kg/m2—a Review	no	can't answer	yes	no	yes	yes	can't answer	yes	yes	can't answer	no	5
29	Impact of roux-en Y gastric bypass surgery on prognostic factors of type 2 diabetes mellitus: meta-analysis and systematic review	no	can't answer	yes	no	yes	yes	can't answer	yes	yes	yes	no	6
30	The Duodenal–Jejunal Bypass Liner for the Treatment of Type 2 Diabetes Mellitus and/or Obesity: a Systematic Review	no	yes	yes	yes	yes	yes	yes	yes	can't answer	can't answer	yes	8
31	[Short- and long-term remission of obese type 2 diabetes mellitus after gastric bypass surgery or sleeve gastrectomy： a Meta analysis]	no	yes	yes	no	yes	yes	yes	yes	yes	can't answer	no	7

32	A Meta-Analysis of Short-Term Outcomes of Patients with Type 2 Diabetes Mellitus and BMI £35 kg/m2 Undergoing Roux-en-Y Gastric Bypass	yes	yes	yes	no	yes	yes	yes	yes	yes	yes	yes	10
33	A systematic review and meta-analysis of the effect of Billroth reconstruction on type 2 diabetes: A new perspective on old surgical methods	no	yes	yes	no	yes	yes	yes	yes	yes	yes	yes	9
34	Bariatric Surgery for Type 2 Diabetes Mellitus in Patients with BMI <30 kg/m2: A Systematic Review and Meta-Analysis	no	yes	yes	yes	yes	yes	yes	yes	yes	can't answer	yes	9
35	Effect of sleeve gastrectomy on type 2 diabetes as an alternative treatment modality to Roux-en-Y gastric bypass: systemic review and meta-analysis	no	yes	yes	no	yes	yes	yes	yes	yes	can't answer	no	7
36	Efficacy of Laparoscopic Mini Gastric Bypass for Obesity and Type 2 Diabetes Mellitus: A Systematic Review and Meta-Analysis	no	yes	yes	no	yes	yes	can't answer	yes	yes	can't answer	no	6

37	Laparoscopic Roux-en-Y Gastric Bypass versus Sleeve Gastrectomy for Obese Patients with Type 2 Diabetes: A Meta-analysis of Randomized Controlled Trials	no	yes	yes	yes	yes	yes	yes	yes	yes	can't answer	no	8
38	Long-Term Effects of Bariatric Surgery on Type II Diabetes,Hypertension and Hyperlipidemia: A Meta-Analysis and Meta-Regression Study with 5-Year Follow-Up	no	yes	yes	no	yes	yes	can't answer	yes	yes	yes	no	7
39	Predictive Factors of Type 2 Diabetes Mellitus Remission Following Bariatric Surgery: a Meta-analysis	no	yes	yes	no	yes	yes	yes	yes	yes	yes	yes	9
40	Predictors of Remission of Diabetes Mellitus in Severely Obese Individuals Undergoing Bariatric Surgery: Do BMI or Procedure Choice Matter? A Meta-analysis	no	yes	yes	no	yes	yes	yes	yes	yes	can't answer	yes	8
41	Surgical Versus Medical Treatment of Type 2 Diabetes Mellitus in Nonseverely Obese Patients： A Systematic Review and Meta-analysis	no	yes	yes	no	yes	yes	can't answer	yes	yes	can't answer	yes	7
42	The Impact of Bariatric Surgery on Diabetic Retinopathy:A Systematic Review and Meta-Analysis	no	yes	yes	no	yes	yes	yes	yes	yes	can't answer	no	7

43	The Long-Term Effects of Bariatric Surgery for Type 2 Diabetes: Systematic Review and Meta-analysis of Randomized and Non-randomized Evidence	no	yes	yes	no	yes	yes	yes	yes	yes	can't answer	yes	8
44	Type 1 Diabetes Mellitus and Bariatric Surgery: A Systematic Review and Meta-Analysis	no	yes	yes	no	yes	no	yes	yes	yes	can't answer	yes	7
45	[Laparoscopic Roux-en-Y Gastric Bypass versus Laparoscopic Sleeve Gastrectomy for Obesity and Type 2 Diabetes Mellitus: A Meta-analysis]	no	yes	yes	no	yes	yes	yes	yes	yes	yes	no	8
46	[Short-Term Efficacy of Laparoscopic Sleeve Gastrectomy for Obesity with Type 2 Diabetes Mellitus in China: A Systematic Analysis]	no	yes	yes	no	yes	yes	yes	yes	yes	yes	no	8
47	Bariatric Surgery in Type 1 Diabetes Mellitus: A Systematic Review	no	yes	yes	yes	yes	yes	can't answer	yes	can't answer	can't answer	yes	7
48	[A META-ANALYSIS OF SLEEVE GASTRECTOMY VERSUS ROUX-EN-Y GASTRIC BYPASS FOR MORBID OBESITY AND DIABETES MELLITUS]	no	yes	yes	no	yes	yes	yes	yes	yes	yes	no	8

49	[Three types of gastrointestinal reconstruction on non-obese type 2 diabetes mellitus： a meta-analysis]	no	can't answer	yes	no	yes	yes	yes	yes	yes	yes	no	7
50	A Systematic Review and Meta-Analysis of Outcomes for Type 1 Diabetes after Bariatric Surgery	yes	can't answer	yes	no	yes	yes	can't answer	yes	yes	can't answer	yes	7
51	Bariatric surgery and diabetic retinopathy_ a systematic review and meta-analysis of controlled clinical studies	yes	can't answer	yes	yes	yes	yes	can't answer	yes	can't answer	can't answer	yes	7
52	Bariatric surgery is associated with less progression of diabetic retinopathyA systematic review and metaanalysis	no	yes	yes	no	yes	yes	yes	yes	yes	yes	no	8
53	Bariatric surgery reduces urinary albumin excretion in diabetic nephropathy_ a systematic review and meta-analysis	yes	yes	yes	yes	yes	yes	yes	yes	yes	yes	yes	11
54	Impact of bariatric surgery on renal functions in patients with type 2 diabetes mellitus systematic review of randomized trials and observational studies	no	yes	yes	yes	yes	yes	yes	yes	yes	yes	no	9
55	Meta-analysis of bariatric surgery versus non-surgical treatment for type 2 diabetes mellitus	no	yes	yes	yes	yes	yes	yes	yes	yes	yes	yes	10

56	Preoperative Fasting Plasma C-Peptide Levels as Predictors of Remission of Type 2 Diabetes Mellitus after Bariatric Surgery: A Systematic Review and Meta-Analysis	no	yes	yes	no	yes	yes	yes	yes	yes	yes	yes	9
57	Roux-En-Y Gastric Bypass in Type 2 Diabetes Patients with Mild Obesity_ a Systematic Review and Meta-analysis	no	can't answer	yes	no	yes	yes	yes	yes	yes	yes	yes	8
58	Roux-en-Y Gastric Bypass Versus Medical Treatment for Type 2 Diabetes Mellitus in Obese Patients_ A Systematic Review and Meta-Analysis of Randomized Controlled Trials	no	yes	yes	no	yes	yes	yes	yes	yes	yes	yes	9
59	Sleeve Gastrectomy and Type 2 Diabetes Mellitus_ a Systematic Review of Long-Term Outcomes	no	yes	yes	yes	yes	yes	can't answer	yes	yes	can't answer	yes	8
60	Type 1 Diabetes Mellitus and Bariatric Surgery_ A Systematic Review and Meta-Analysis	no	can't answer	yes	no	yes	yes	yes	yes	yes	can't answer	yes	7
61	[Meta-analysis of traditional treatment and weight loss surgery in the clinical treatment of obese type 2 diabetes patients]	no	can't answer	yes	no	yes	no	yes	yes	can't answer	can't answer	no	4

62	[A Meta-analysis of therapeutic effect and related complications of laparoscopic sleeve gastrectomy and laparaoscopic Roux-en-Y gastric bypass]	no	yes	yes	no	yes	yes	yes	yes	yes	yes	no	8
63	[The Short-Term Effect Analysis of Laparoscopic Sleeve Gastrectomy and Laparoscopic gastric bypass for obesity and type 2 diabetes（T2DM）in China]	no	yes	yes	yes	yes	yes	yes	yes	yes	yes	no	9
64	[Comparative on clinical effect and cost-effectiveness Bariatric Surger for patients with type 2 Diabetes Mellitus]	no	can't answer	yes	no	yes	yes	yes	yes	yes	yes	no	7
		5	51	61	13	60	59	45	64	55	28	31	
